# The Importance of Measuring SARS-CoV-2-Specific T-Cell Responses in an Ongoing Pandemic

**DOI:** 10.3390/pathogens12070862

**Published:** 2023-06-22

**Authors:** Linda Petrone, Alessandro Sette, Rory D. de Vries, Delia Goletti

**Affiliations:** 1 Translational Research Unit, National Institute for Infectious Diseases “Lazzaro Spallanzani”-IRCCS, 00149 Rome, Italy; linda.petrone@inmi.it; 2 Center for Infectious Disease and Vaccine Research, La Jolla Institute for Immunology (LJI), La Jolla, CA 92037, USA; alex@lji.org; 3 Department of Medicine, Division of Infectious Diseases and Global Public Health, University of California, San Diego (UCSD), La Jolla, CA 92037, USA; 4 Department Viroscience, Erasmus University Medical Center, 3015CN Rotterdam, The Netherlands; r.d.devries@erasmusmc.nl

**Keywords:** humoral immunity, cellular immunity, T-cell response, interferon-γ release assay, SARS-CoV-2, vaccination, immunocompromised

## Abstract

Neutralizing antibodies are considered a correlate of protection against SARS-CoV-2 infection and severe COVID-19, although they are not the only contributing factor to immunity: T-cell responses are considered important in protecting against severe COVID-19 and contributing to the success of vaccination effort. T-cell responses after vaccination largely mirror those of natural infection in magnitude and functional capacity, but not in breadth, as T-cells induced by vaccination exclusively target the surface spike glycoprotein. T-cell responses offer a long-lived line of defense and, unlike humoral responses, largely retain reactivity against the SARS-CoV-2 variants. Given the increasingly recognized role of T-cell responses in protection against severe COVID-19, the circulation of SARS-CoV-2 variants, and the potential implementation of novel vaccines, it becomes imperative to continuously monitor T-cell responses. In addition to “classical” T-cell assays requiring the isolation of peripheral blood mononuclear cells, simple whole-blood-based interferon-γ release assays have a potential role in routine T-cell response monitoring. These assays could be particularly useful for immunocompromised people and other clinically vulnerable populations, where interactions between cellular and humoral immunity are complex. As we continue to live alongside COVID-19, the importance of considering immunity as a whole, incorporating both humoral and cellular responses, is crucial.

## 1. Introduction

Virus-specific (neutralizing) antibodies and memory immune cells (acquired naturally or through vaccination) play complementary roles in responding to SARS-CoV-2 infection and protecting against COVID-19 [1,2].

Currently approved COVID-19 vaccines all target the SARS-CoV-2 spike (S) protein [3,4]. S-targeting vaccines have been shown to induce both antibody and T-cell responses [2], although the magnitude of the response varies between different vaccines [1,5]. Neutralizing antibodies have been identified as a correlate of protection [6]; concomitantly, a robust, timely, and coordinated adaptive CD4+ and CD8+ T-cell response may be critical for attenuating the severity of COVID-19 [1,7,8,9].

As we continue to live alongside COVID-19, the need to comprehensively evaluate the characteristics of the adaptive immune response to SARS-CoV-2 is becoming more pertinent, particularly with the emergence of antigenically distinct variants, which have the propensity for (partial) escape from neutralizing antibodies [2,5,8]. It is imperative that we learn more about the durability and dynamics of immune responses, including the virus-specific T-cell response, to identify the extent to which vaccines retain effectiveness against variants and to inform vaccination policies and booster programs [8,10]. Virus-specific T-cells are an essential consideration for understanding and mitigating the high burden of COVID-19 in vulnerable populations, such as the elderly or immunocompromised, where inadequate immune responses may worsen patient outcomes [9,10,11,12].

This review highlights the significance of monitoring SARS-CoV-2-specific T-cell responses and their potential long-term role in protecting against severe COVID-19. The importance of considering both cellular and humoral immunity is underscored, and evidence is presented to support the measurement of T-cell responses as a valuable tool to evaluate immunity.

## 2. Current Views on SARS-CoV-2-Specific T-Cell Responses

### 2.1. T-Cell Responses following SARS-CoV-2 Infection and COVID-19 Vaccination

Effective viral clearance and mild COVID-19 following SARS-CoV-2 infection are associated with an early innate immune response [1,13] followed by a virus-specific CD8+ T-cell response (within 7 days of symptoms and peaking after 14 days, mirroring antibody kinetics) and mobilization of CD4+ T helper type 1 cells [14]. Both CD8+ and CD4+ T-cells show considerable expansion within 4 weeks [14] and detectable levels can be maintained for at least 6–8 months [14,15]. COVID-19 vaccines based on mRNA, adenovirus vector, or inactivated virus platforms and validated for use in humans [4] also induce SARS-CoV-2-specific CD8+ and CD4+ T-cell responses [14,16] that resemble the timing of responses following natural infection [17,18]. However, following (mRNA-based) vaccination, the early memory pool of CD8+ T-cells appears distinguishable from that of natural infection with regard to memory T-cell subsets distribution, possibly due to differences in the location or duration of contact with the antigen, or inflammatory responses following vaccination versus infection [18]. With the exception of inactivated vaccines [19] other approved vaccines to date rely solely on the targeting of the S antigen and, thus, generate only S-specific memory responses, whereas in most convalescent individuals, S-specific T-cells represent a minority of the cellular populations [20]. In addition to S-specific responses, a broader repertoire of T-cell reactivity may have relevance in vaccine-derived protection against SARS-CoV-2 by targeting other T-cell inducing components such as the nucleocapsid (N) protein, nonstructural protein (NSP) antigens encoded in the open reading frames (ORFs) of the genome, or the antigen domains of fragments that have abundant T-cell epitopes [21,22,23].

### 2.2. Durability of SARS-CoV-2-Specific T-Cell Responses

**Figure 1:**
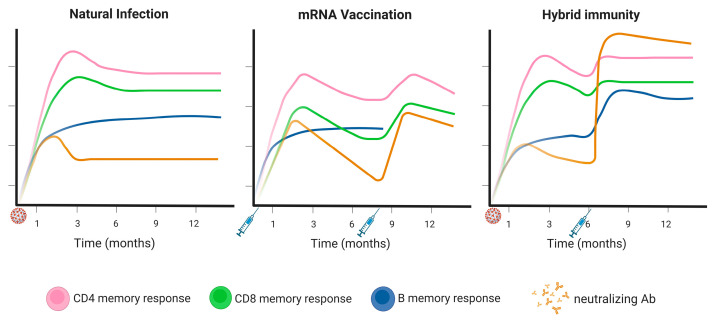
Durability of the memory response to SARS-CoV-2. The different components of the memory response to SARS-CoV-2 after natural immunity, mRNA vaccination, or hybrid immunity show different kinetics defining the durability of the response and, therefore, the protection against severe disease and breakthrough infections. The scales are not quantitative. The CD4 or CD8 memory response is intended to spike for the mRNA vaccination and to the entire virus for the infection. The B memory and neutralizing responses are intended to spike. The infection is represented by SARS-CoV-2. In the “mRNA vaccination” plot, the booster dose is considered at 8 months. In hybrid immunity, the vaccination is considered at 6 months. In both cases, the vaccination is represented by a syringe. Footnotes: mRNA: messenger ribonucleic acid; Ab: antibody. Created with Biorender.com.

Data are rapidly accumulating on the durability and dynamics of SARS-CoV-2-specific immune responses (Figure 1). Evidence suggests that patients who previously had SARS in the 2003 outbreak (the disease associated with SARS-CoV infection) maintained memory T-cell reactivity to the N protein of the virus for 17 years, suggesting a long-term durability of T-cell responses [24].

Following SARS-CoV-2 infection, large population-based studies have shown that, while circulating antibody levels are well maintained for at least 3–4 months [25,26], CD4+ and CD8+ T-cells can persist for longer (at least 6–8 months) [15].

After two-dose vaccination regimens, T-cell responses occur within 28 days and persist for at least 6 months [8,16]. S-specific and neutralizing antibody responses to a third or “booster” vaccine are also detectable at 5 months post-booster vaccination, but at a lower level compared with those seen at 28 days [27]. Therefore, despite the obvious waning of the antibody response over time, vaccination induces the formation and persistence of T-cell immunity [28]. Following a booster vaccine dose, levels of neutralizing antibodies to SARS-CoV-2 appear potentiated compared with responses following two doses; T-cell reactivity can also be augmented by a booster dose, peaking within 2 weeks [5,29,30,31,32], which may reflect the long-term persistence of earlier vaccine-induced T-cell responses. The extent of the effect of boosters on both humoral and cellular responses appears to be vaccine- and variant-specific [5,29,31,32].

### 2.3. Hybrid Immunity and Breakthrough Infections

Now that SARS-CoV-2 has been circulating worldwide for more than 3 years, the heightened and robust protection that is afforded by a combination of naturally acquired infection and vaccination (i.e., “hybrid immunity”) has become increasingly apparent, as supported by immunological and epidemiological evidence [31].

In individuals with SARS-CoV-2 infection prior to vaccination, CD4+ T-cells were detected in the convalescent phase and were boosted after a first mRNA-based vaccine dose, with a second dose offering no additional boosting effect [2]. CD8+ T-cells were also present following recovery from COVID-19 and were increased following two mRNA-based vaccination doses [2] (Figure 1). Additionally, prior COVID-19 promoted the development of high levels of neutralizing antibodies and antibody-dependent cellular cytotoxicity (ADCC)-mediating responses following a single vaccination, which were not observed in COVID-19-naïve individuals until after the second vaccination dose [2]. It has also been shown that being infected during the first (ancestral virus) or second (Beta variants) wave of COVID-19 in South Africa prior to adenovirus-vector-based vaccination boosted S-specific binding antibodies, neutralizing antibodies, and ADCC, and moderately boosted CD4+ and CD8+ T-cell responses [33]. Further, neutralizing antibody responses to mRNA- or adenovirus-vector-based vaccines were higher in healthcare workers who had previously been infected with SARS-CoV-2 than those who were naïve to infection [5]. These studies highlight an enhanced response to vaccination from the priming of the immune system by prior SARS-CoV-2 exposure.

Similarly, hybrid immunity can be acquired from the priming of the immune system by vaccination followed by subsequent natural infections. These breakthrough infections can depend on several factors related to both the host and virus [34,35]. Whereas antibody responses are elicited by breakthrough infections [35], the available data on T-cell responses are more complex to interpret. Indeed, it was shown that SARS-CoV-2 infection after spike-based vaccination allows the development of T-cells specific against other SARS-CoV-2 antigens [36], and a rapid and extensive recall of spike-specific CD4 and CD8 occurs early after Delta or Omicron breakthrough infection [37]. Moreover, several studies show that T-cell frequencies do not differ between SARS-CoV-2 breakthrough infections and non-breakthrough cases [38,39], with enhanced spike-specific T-cells in some reports [40]. In contrast, mRNA-vaccinated individuals who experienced severe COVID-19 as consequence of a breakthrough infection had a delayed T-cell response to S [41]. Monitoring breakthrough infections is important to guide the development of novel vaccines, especially in the current scenario where antigenically distinct variants have emerged.

### 2.4. T-Cell Responses as a Potential Correlate of Protection

There is a crucial role for neutralizing antibodies in vaccine-induced protection from infection [6], while T-cells could have potential in limiting disease severity [42]. Indeed, clinical outcomes in COVID-19 are at least partly determined by the functional capacity of T-cell responses: efficient viral clearance and mild disease are associated with a rapid induction of CD4+ and CD8+ T-cells, whereas severe disease and fatal outcomes are more likely in the absence of these responses [1,9,14]. In contrast, the presence of neutralizing antibodies alone is insufficient to control disease [9]. In convalescent rhesus macaques, the depletion of CD8+ T-cells partially abrogated the protective efficacy of natural immunity against rechallenge with SARS-CoV-2, suggesting a role for T-cell immunity in the context of waning or subprotective antibody titers [43]. A separate study showed that vaccine-elicited CD8+ T-cells contributed substantially to virologic control following SARS-CoV-2 challenge in rhesus macaques, with CD8-depleted animals showing higher viral levels in the upper and lower respiratory tract than non-CD8-depleted animals [44]. Interestingly, the SARS-CoV-2-specific CD4+ T-cell response appears to have the dominant protective role for lessening COVID-19 severity and controlling and clearing infections [9].

### 2.5. T-Cell Immunity in Specific Populations

**Figure 2:**
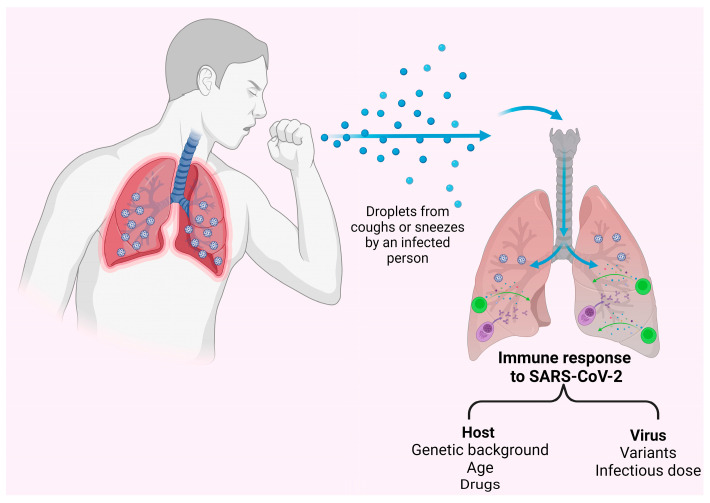
Factors impacting the immune response to SARS-CoV-2. The development of the adaptive immune response to SARS-CoV-2 may depend on host factors or viral features. The immune “status” of an individual at any moment (i.e., primary immunodeficiency or immune-mediated disorders), the age, and/or immunomodulating drugs are well recognized host factors that may have an impact on the induction and durability of both B and T-cell response to SARS-CoV-2. Moreover, viral factors as the emergence of new viral variants of concern, with higher degree of immune escape and infectivity, as well as the level of exposure to SARS-CoV-2, may be associated with a lesser immune protection against sever COVID-19 and breakthrough infections. Footnotes: SARS-CoV-2: severe acute respiratory syndrome coronavirus 2. Created with Biorender.com.

**Figure 3:**
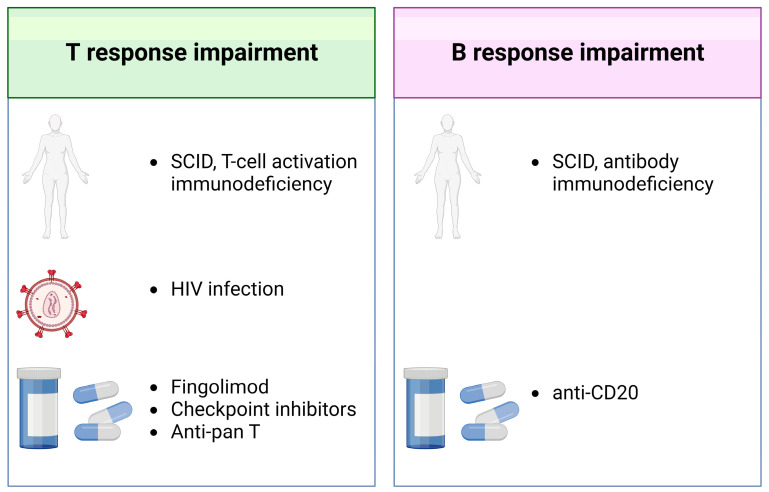
Principle causes of impairment of T- and B-cell immunity for responding to SARS-CoV2 infection. The immune host background (i.e., inborn errors of immunity), infections, and several drugs may differently modify SARS-CoV-2-specific response. Known causes of T-cell response impairment are combined (SCID) as well as T-cell activation immunodeficiency syndromes, HIV infection, and drugs such as fingolimod or checkpoint inhibitors and anti-Pan T, used for immune-mediated disorders or malignancies. Similarly, B-cell response impairment may be attributed to SCID or antibody deficiencies and drugs that affect B-cell functions as anti-CD20. Footnotes: SCID: severe combined immunodeficiency; HIV: human immunodeficiency virus. Created with Biorender.com.

In clinically vulnerable individuals, the interaction between adaptive and humoral immunity is often atypical and complex, and there are varying degrees of antibody and T-cell responses to natural infection and vaccination depending on several factors [1,9,11,42,45,46,47,48,49,50,51,52,53,54] (Figure 2 and Figure 3).

### 2.6. Elderly

Individuals who are older than 65 years of age have a higher risk of developing severe COVID-19. This may be due to low frequencies of naïve T-cells [35,36] and, therefore, to a scarcity of T-cells able to respond to new antigens. Moreover, in older people, SARS-CoV-2 infection contributes to the loss of a coordinated response between the cellular and the humoral responses. The CD8 effector response mediated by granzyme and perforin is also reduced in elderly people older than 80 years of age [9]. The evidence that age impairs T-cell immunity with an impact on controlling infections is also available for other diseases, like AIDS and tuberculosis [55].

### 2.7. People with Immune-Mediated Disorders

Patients with immune-mediated disorders face a higher risk of severe disease or even death from COVID-19 and are more likely to mount a delayed immune response or produce insufficient SARS-CoV-2-specific antibodies [1,11,45,54,56]. Patients with immune-mediated inflammatory diseases mount an immune response to SARS-CoV-2, even when infected with viral variants [49,57,58]; they also generate a specific response after vaccination [56]. However, this response may have a lower intensity and less durability compared with controls, mainly in those taking T-cell-targeted or B-cell-targeted therapies [53,59]. Similarly, in patients with multiple sclerosis undergoing immune-suppressive treatments, several studies reported a low or absent humoral- and cell-mediated immunity [60]; booster mRNA vaccine doses reinforce specific immunity, although this is dependent on the type of therapy used [61]. In particular, patients receiving CD20 inhibitors may fail to develop a sufficient antibody response to COVID-19 vaccination. In addition, patients treated with fingolimod, a disease-modifying therapy for multiple sclerosis that reduces T-cell egress from the lymph nodes and reduces the levels of circulating lymphocytes [50,62,63], have a blunted antibody- or T-cell-mediated response to COVID-19 vaccination. Importantly, whether T-cell responses are able to protect patients with immune-mediated disorders from severe disease is still matter of debate [35]. In particular, fingolimod use does not appear to be related to a greater risk of severe COVID-19 [64], suggesting an ongoing protective role of immune responses in the lymphoid tissues [65]. However, the retention of T-cell responses postvaccination [50,62], particularly in the absence of functional antibodies [66], is important for protection, and highlights the need to consider immunity as a whole (both humoral and cellular).

### 2.8. People with Primary Immunodeficiencies

Whereas the majority of subjects with primary immunodeficiencies, or inborn errors of immunity (IEI), undergo a mild course of COVID-19, people with some specific forms of IEI, as combined immunodeficiencies, antibody defects (i.e., X-linked agammaglobulinemia) or NF-kB deficiency, showing an impairment of the adaptive immune responses may fail to control SARS-CoV-2 infection and may be at higher risk of developing severe COVID-19 [67,68]. In this context, it is also important to understand the efficacy of COVID-19 vaccines. In particular, it has been shown that patients with IEI are able to mount both a humoral and cellular response [69,70]). The possibility of detecting a vaccine-induced T-cell response, which may reduce disease severity, also in patients who lack B-cells suggests that patients with IEI could still benefit from vaccination [69,70].

### 2.9. PLWH

People living with HIV (PLWH) are considered at high risk of severe COVID-19, mainly in the case of low CD4+ counts [71] or unsuppressed viremia. Antiretroviral therapy, suppressing the viral load, may play an important role in the development of a robust T-cell response. Indeed, it has been demonstrated that SARS-CoV-2-specific CD4+ and CD8+ T-cell responses are detectable in PLWH with controlled HIV infection [72]. T-cell responses are also detectable in mRNA-vaccinated HIV patients. However, the magnitude of the response is reduced in patients with a CD4+ T-cell count < 200 cells/µL [73]. Vaccine-induced responses persist up to six months after vaccine schedule completion, even if a slight decline was observed over time [74]. Moreover, whereas antibodies titers are increased by boosters, the T-cell responses seem to be unaffected [75].

### 2.10. Solid Organ Transplant Recipients

Solid organ transplant recipients were able to mount a SARS-CoV-2-specific T-cell response after vaccination or infection. This response seems to be qualitatively and quantitatively similar to that observed in controls [74]. However, the induction and the maintenance of the T-cell responses are influenced by the disease severity [76]. Like other vulnerable populations, the increased risk for severe COVID-19 comes from the treatment of solid organ transplant recipients with immunomodulating drugs. Therefore, vaccination strategies in these patients should be carefully evaluated. Indeed, several studies demonstrated an impaired CD4 and CD8 T-cell response, and more importantly, an attenuated antibody response after SARS-CoV-2 vaccination [77]. Moreover, therapies may profoundly affect the vaccine-induced response. In particular, an impairment of both humoral and cellular response has been shown in allogeneic hematopoietic stem cell transplant (allo-HSCT) recipients taking corticosteroids during or prior the vaccination administration [78]. Vaccine-induced T-cell response is also influenced by the time between vaccine administration and transplant, with effector memory CD4 T-cells being detectable after CD4 reconstitution [78]. T-cell response was also increased through completion of the vaccine schedule in allo-HSCT patients. Indeed, the rate of T-cell responders increased from 35.3% (after the first dose) to 82.3% (after the second dose) [79]. Due to the uncertainty of the persistence of vaccine-induced immune response, it has been recommended that patients with HSCT can receive a fourth vaccine booster [80].

### 2.11. Solid and Hematologic Cancer Patients

Patients with cancers have higher COVID-19 morbidity and mortality, mainly when the elderly or patients with comorbidities are infected with SARS-CoV-2. It has been extensively documented that patients with solid tumors have a sustained antibody response and higher frequencies of virus-specific CD4 and CD8 compared to hematological malignances [81]. Moreover, these patients with hematologic malignancies show high expression of T-cell exhaustion markers [81]. This immune impairment further highlights the importance of the T-cell response in protecting from severe disease. COVID-19 vaccines induce a low antibody response, mainly in patients with hematologic disorders, and a reduced T-cell response, that was similar between solid and hematologic cancers [74,82]. Like in immune-mediated disorder, therapies with CD20 inhibitors may drastically impair the antibody response in patients with hematologic malignancies [83]. The timing of CD20 inhibitors therapies is an important factor to consider for vaccine-induced response. Indeed, it has been shown that if COVID-19 vaccination is performed during the treatment, the rate of seroconversion is not impacted; on the other hand, if vaccination is performed after treatment completion or within 12 months, an improvement from 40 to 70% is observed [84]. In contrast, vaccine-induced T-cell response is less impaired by CD20 inhibitors [83]. Indeed, even if the T-cell response is observed mainly in seroconverted patients [85] and was less associated with the time from the last CD20 inhibitors dose administration [84], it may be detected even in the absence of a detectable humoral response [74], supporting the benefit of vaccination even in the case of these therapies. Moreover, like patients with HSCT, booster vaccination doses have been suggested for patients with hematologic malignancies [80]. In contrast to CD20 inhibitors, anticancer therapies with checkpoint inhibitors seem to be associated with an impaired T-cell response, mainly in the CD4 compartment [78].

Combined, all this evidence highlights that immune fragilities require tailored clinical strategies and immunocompromised patients should have access to primary prophylaxis [86], early SARS-CoV-2 detection, and prompt and proper management of COVID-19.

## 3. Immune Responses to Emerging SARS-CoV-2 Variants

While vaccines were crucial to protect against severe COVID-19 and mortality early in the pandemic (and continue to be so), it remains unclear whether it is necessary to boost the existing immune response in communities where SARS-CoV-2 infections are commonplace and there is pre-existing immunity from both vaccination and infection. The emergence of novel variants, particularly the Omicron sublineages at the time of writing, which have a high degree of humoral immune escape and infectivity compared with other variants and a propensity to cause repeated infections, complicates the question about the necessity for continuous booster vaccinations [5,87].

In individuals receiving a course of approved mRNA- or adenovirus-vector-based vaccines or a whole inactivated virus vaccine, antibody reactivity to SARS-CoV-2 is considerably reduced for variants, including Beta, Gamma, Delta, and Omicron, compared with the ancestral strain [2,4,5,16,33,88,89,90,91]. Diminished humoral responses to variants have also been reported in COVID-19 convalescent individuals or those previously infected with SARS-CoV-2 and later vaccinated [16,88,90,91]. Low cross-reactivity of neutralizing antibodies is reported for the Omicron sublineages, reflecting high numbers of mutations and deletions in the S protein, including in the receptor binding domain, essential to gain host cell entry [16]. Booster vaccinations (i.e., third or fourth doses) in general restored the antibody cross-neutralization of Omicron variants, with mRNA-based vaccines appearing to be more effective than adenovirus-vector-based vaccines [5,16,92]. Frequent boosters could be necessary for vulnerable populations with inadequate immune responses to vaccination to help sustaining protective immunity [93,94].

In contrast to the detrimental effects of variants on antibody reactivity, it is encouraging that polyclonal T-cell responses to SARS-CoV-2 following vaccination and/or infection are largely maintained, despite the abundance of mutations, even in the case of Omicron [2,8,16,20,33,42,95]. A detailed cohort study of COVID-19 vaccine recipients reported that variant-specific memory T-cell responses are preserved across vaccine platforms (both mRNA and adenovirus-vector-based) for up to 6 months postvaccination [16]. Minimal immune escape to SARS-CoV-2 variants at the T-cell level may provide an additional line of defense to help counteract the low cross-reactivity of neutralizing antibodies and protect against severe COVID-19 [30]. Recent data suggest that T-cell reactivity to Omicron can be boosted following a third vaccine dose [30].

Differently to neutralizing antibodies, it is hypothesized that T-cells are reactive to emerging variants because of their ability to recognize a wider range of epitopes [8,20,96]. The vast majority of T-cell epitopes (including epitopes in the S protein) are conserved in variants, and T-cell affinity appears to be unaffected by variant mutations [8,20,97]. Overall, polyclonal SARS-CoV-2 T-cell reactivity to and recognition of variants appear to be only modestly reduced in vaccinated and COVID-19-recovered individuals [8,95], even if certain T-cell clones targeting specific mutated epitopes may lose reactivity [95]. In addition, vulnerable populations such as those with immune-mediated inflammatory disease [49] or multiple sclerosis [12,50] still show intact T-cell responses and retain the ability to recognize variants, even though they are receiving immunosuppressive drugs.

## 4. T-Cell Vaccines

As T-cells can recognize conserved viral epitopes, vaccines aimed at the specific induction of virus-specific T-cells might provide even broader reactivity to SARS-CoV-2 variants [97]. Early studies aiming to identify suitable SARS-CoV-2 epitopes to target with vaccines identified strong CD4+ and CD8+ T-cell responses to the membrane (M) protein; N protein; NSP3, 4, 6, 7, 12, and 13 (ORF1ab); and ORF3a and ORF8, in addition to the S protein [42,98,99]. As some of the most dominant SARS-CoV-2-specific CD8+ T-cell responses are directed against non-S epitopes, extending vaccines to non-S antigens would increase the breadth of T-cell responses even further [20]. Several vaccines with multiple targets (more than one S protein (e.g., bivalent vaccines), or including antigens other than the S protein) to induce broad immune responses are currently in preclinical and clinical trials; these include mRNA-based, protein-based, DNA-based, and viral-vector-based platforms [96,100]. Developments in the field of T-cell vaccines might be key to protecting against antigenically distinct variants that can potentially overcome immunity induced by current vaccines [97].

## 5. Potential Role and Value of Whole-Blood-Based Interferon (IFN)-γ Release Assays (IGRAs) in Immune Monitoring

### Methods and Considerations for Measuring T-Cell Responses

Detecting virus-specific T-cell responses can help to better understand how vaccines protect against SARS-CoV-2 infection and the development of severe COVID-19. Both the longevity of that protection and the reactivity of immune responses with variants are crucial pieces of information to determine vaccine policy, including the optimal frequency of booster vaccination, particularly in high-risk populations [10,45,47]. At the molecular level, T-cell responses can be investigated using next-generation sequencing platforms to sequence the T-cell receptor DNA, although technical challenges, including analyses, and costs have limited the adoption of this technology outside of research laboratories [10]. The detection of antigen-specific immune responses on the cellular level includes the enzyme-linked immunosorbent spot (ELISpot) assay, intracellular cytokine staining (ICS), or the activation-induced marker (AIM) assay, which predominantly examine the T-cell recall response in (cryopreserved) peripheral blood mononuclear cells (PBMCs) isolated from blood [10]. The ELISpot assay is relatively easy and inexpensive to employ, although it provides limited information on the phenotype of antigen-experienced cells. The ICS and AIM approaches tackle this shortcoming but require costly equipment and a degree of specialist training to ensure that the measurement of related cytokines or surface antigen markers is correctly characterized and linked to T-cell phenotypes [10].

A simpler alternative method for detecting T-cell responses is provided by functional cellular assays that are based on the detection of excreted IFN-γ as an established blood-based marker of T-cell activation (i.e., IGRAs) [101]. IGRA assessment of the S-specific T-cell response from fresh whole blood shows high correlation with the results obtained with traditional assays (including AIM and ELISpot) [101], although the sensitivity of different assays may vary in patients with underlying conditions [102]. Although not yet approved for diagnostic use in the context of COVID-19 natural infection and vaccination, IGRAs are well recognized tools for the detection of Mycobacterium tuberculosis infection [10,103]. IGRAs have been widely used in studies of COVID-19 patients [104,105] and to investigate T-cell responses to COVID-19 vaccination [101,106]. IGRAs have also been used postvaccination to evaluate the nature of T-cell responses in other studies of healthcare workers; individuals with low versus high humoral responses; and patients who are immunosuppressed, immunocompromised, on hemodialysis, or have coinfections [45,48,98,107,108,109,110,111,112].

Measuring T-cell responses in whole blood using IGRAs is a straightforward procedure with short turnaround times, and has the added advantage of more closely reflecting in vivo conditions than testing purified PBMCs [113]. However, it may not accurately reflect the multifaceted nature of total immunity (also incorporating humoral immunity and the contribution of memory B-cells) [1]. In addition, the absence of detectable T-cell activity in the blood does not necessarily equate to the absence of virus-specific T-cells from lymphoid tissues, in which these cells may be readily reactivated in response to infection or vaccination [114]. Indeed, fingolimod treatment in patients with multiple sclerosis is characterized by the sequestration of T-cells in lymphoid tissues and low T-cell S-specific response in the peripheral blood [50], yet the risk of severe COVID-19 appears to be similar to that of the general population or the multiple sclerosis population overall [64]. Despite their limitations, because IGRAs are characterized by ease of use and an ability to accurately evaluate the magnitude and monitor T-cell response, they may help clarify the picture of T-cell responses against SARS-CoV-2, particularly as an adjunct to other immune response investigations.

## 6. Conclusions and Future Directions

A mounting body of evidence points to the importance of evaluating T-cell responses alongside humoral responses when assessing the protective effects of vaccines and predicting outcomes following SARS-CoV-2 infection on an individual basis, particularly for those at greater risk of severe COVID-19.

Questions remain regarding the degree to which T-cells contribute to protective immunity and the longevity of these responses. The concept of hybrid immunity resulting from a combination of a natural infection and vaccination is becoming more relevant as SARS-CoV-2 continues to circulate. This leads to additional questions regarding the necessity of continuous booster vaccinations for the general population, and at which frequency these are given, to promote both optimal humoral and cellular responses. Additionally, sufficient research regarding which vaccines may be most effective in priming, and in particular boosting, T-cell responses is lacking.

A thorough evaluation of immune responses to inactivated virus- or protein-based vaccines has yet to be performed. These questions are further complicated by the evolution of SARS-CoV-2, as a significant detrimental impact of variants on antibody reactivity, in particular, has been observed. It is clear that T-cell responses are robust in protecting against severe COVID-19, including disease caused by SARS-CoV-2 variants, even in patients who are immunocompromised or otherwise clinically vulnerable. Although T-cell response monitoring in clinical practice is not yet routinely employed, evidence to support the value of simple assays that could be implemented diagnostically, such as IGRAs, is accumulating in the research setting. The widespread use of such assays could help us to advance our understanding of the T-cell response to SARS-CoV-2 infection and/or COVID-19 vaccination, contributing to the development of new vaccines (for example, T-cell-based vaccines targeted at conserved viral epitopes) and guiding decisions on vaccine booster programs as we learn to live alongside COVID-19.

## Data Availability

Not applicable.

## Abbreviations

ADCC: antibody-dependent cellular cytotoxicity; AIM: activation-induced marker; ELISpot: enzyme-linked immunosorbent spot; ICS: intracellular cytokine staining; IFN: interferon; IGRA: IFN-γ release assay; M: membrane; N: nucleocapsid; NSP: nonstructural protein; ORF: open reading frame; PBMC: peripheral blood mononuclear cell; S: spike.
